# Fetal birthweight prediction with measured data by a temporal machine learning method

**DOI:** 10.1186/s12911-021-01388-y

**Published:** 2021-01-25

**Authors:** Jing Tao, Zhenming Yuan, Li Sun, Kai Yu, Zhifen Zhang

**Affiliations:** 1 Department of Obstetrics and Gynecology, The Affiliated Hangzhou People’s Hospital of Nanjing Medical University, Hangzhou, China; 2 Engineering Research Center of Mobile Health Management Ministry of Education, Hangzhou Normal University, Hangzhou, China; 3 Department of Obstetrics and Gynecology, Hangzhou Women’s Hospital, Hangzhou, China; 4 Department of Research and Development, Hangzhou Hele Tech.Co, Hangzhou, China

**Keywords:** Fetal birthweight prediction, Health data mining, Pregnant healthcare, Temporal data mining

## Abstract

**Background:**

Birthweight is an important indicator during the fetal development process to protect the maternal and infant safety. However, birthweight is difficult to be directly measured, and is usually roughly estimated by the empirical formulas according to the experience of the doctors in clinical practice.

**Methods:**

This study attempts to combine multiple electronic medical records with the B-ultrasonic examination of pregnant women to construct a hybrid birth weight predicting classifier based on long short-term memory (LSTM) networks. The clinical data were collected from 5,759 Chinese pregnant women who have given birth, with more than 57,000 obstetric electronic medical records. We evaluated the prediction by the mean relative error (MRE) and the accuracy rate of different machine learning classifiers at different predicting periods for first delivery and multiple deliveries. Additionally, we evaluated the classification accuracies of different classifiers respectively for the Small-for-Gestational-age (SGA), Large-for-Gestational-Age (LGA) and Appropriate-for-Gestational-Age (AGA) groups.

**Results:**

The results show that the accuracy rate of the prediction model using Convolutional Neuron Networks (CNN), Random Forest (RF), Linear-Regression, Support Vector Regression (SVR), Back Propagation Neural Network(BPNN), and the proposed hybrid-LSTM at the 40th pregnancy week for first delivery were 0.498, 0.662, 0.670, 0.680, 0.705 and 0.793, respectively. Among the groups of less than 39th pregnancy week, the 39th pregnancy week and more than 40th week, the hybrid-LSTM model obtained the best accuracy and almost the least MRE compared with those of machine learning models. Not surprisingly, all the machine learning models performed better than the empirical formula. In the SGA, LGA and AGA group experiments, the average accuracy by the empirical formula, logistic regression (LR), BPNN, CNN, RF and Hybrid-LSTM were 0.780, 0.855, 0.890, 0.906, 0.916 and 0.933, respectively.

**Conclusions:**

The results of this study are helpful for the birthweight prediction and development of guidelines for clinical delivery treatments. It is also useful for the implementation of a decision support system using the temporal machine learning prediction model, as it can assist the clinicians to make correct decisions during the obstetric examinations and remind pregnant women to manage their weight.

## Background

The fetal birthweight is an important indicator of the prognosis of the perinatal health. The correct prediction of the birthweight is undoubtedly of great significance to determine the appropriate delivery method. It will be very important to reduce the occurrence of babies who are overweight or under gestational age and is also critical for both short-term and long-term health outcomes in neonates. Being born small for gestational age (SGA) is associated with seizures, respiratory distress, hypoglycemia, hyperbilirubinemia, polycythemia, thrombocytopenia and necrotizing enterocolitis. [1] On the other hand, the perinatal morbidity associated with large for gestational age (LGA) infants is related to a prolonged and complicated labor due to the physical size and includes birth injury, the need for operative vaginal delivery or caesarean section, asphyxia and meconium aspiration. Other postnatal problems that are commonly seen in LGA infants are hypoglycemia, hyperbilirubinemia, polycythemia and respiratory distress. On average, compared with neonates born appropriate for gestational age (AGA), SGA and LGA infants are more likely to need extra medical care during the delivery admission and readmission within two weeks of delivery. Therefore, estimating the birthweight during pregnancy helps to determine whether the fetal development is normal. Additionally, it has a guiding role in selecting the delivery mode during late pregnancy.

However, the birthweight cannot be directly measured before delivery and is usually roughly estimated according to the experience of the clinicians. Most empirical formulas come from applying regression analysis on the B-ultrasound measurement results of pregnant women. Shepard et al. [2] directly calculated the birthweight using parameters such as the biparietal diameter (BPD) and abdominal circumference (AC). Hadlock et al. [3] used the factors of head circumference (HC), abdominal circumference (AC) and femur length (FL) to predict the birthweight via regression analysis. Zhu et al. [4] compared the accuracy of six empirical formulas using the uterine height and abdominal circumference and found the highest accuracy in the six empirical calculations to be 45.76%, which does not meet the current clinical needs. Möstl et al. [5] considered that the traditional empirical formulas were based on the results of a single-point prediction, which made it easy to explain its significance but omitted the measurement of the uncertainty of the prediction intervals. Therefore, they proposed using the uncertainty to improve the robustness of the conditional linear transformation model in predicting the birthweight. Hong et al. [6] mentioned that the empirical formulas used in the clinical practice were mostly established for different areas. For example, there are racial differences among different ethnic groups in the measurement results. Therefore, appropriate adjustments need to be made to the empirical formulas’ methods to adjust them for the specific circumstances. Furthermore, due to the differences in the maternal self-parameters and measurement methods, it is very difficult to establish a general empirical calculation, which leads to the low accuracy of the empirical prediction calculations [7].

Machine learning (ML) technologies have been recently used to predict the birthweight. Farmer et al. [8] firstly proposed an artificial neural network (ANN) for fetal body weight prediction using the results of the B-ultrasound to include the physical characteristics of pregnant women. They predicted the birthweight using a BP neural network (BPNN) according to the parameters of BPD, HC, AC, FL, amniotic fluid index time, birth, height and others. The results showed the BPNN to be better than the traditional regression analysis. Cheng et al. [9] proposed a clustering-based ANN model for birthweight prediction. Mohammadi et al. [10] used an ANN to predict the weight of twin fetuses. Feng M [11] used an SVM and deep belief network (DBN) ML solution to improve the fetal weight estimation accuracy and to help the clinicians identify potential risks before delivery. Kuhle et al. [7] referred to the classification concept (SGA, AGA, LGA) of the birthweight with actual clinical values and compared and analyzed the prediction accuracy of the logarithmic weight classification (LR) and ML methods for different pregnancies. However, the above-mentioned prediction models ignored the effect of time-dependent factors.

In fact, the fetal birthweight is closely related to pregnant woman’s pre-pregnancy weight and weight changes during pregnancy. Since the weight changes are time series data, this paper proposes a time series birthweight prediction model based on the long short-term memory (LSTM) networks. The model is trained on a large dataset of real maternal data, which is composed of the historical obstetric examinations and pregnancy outcomes of 5,759 pregnant women in China. The experimental results show that the hybrid prediction model improved the prediction accuracy by 14% compared with the traditional ML prediction method. Compared with the BPNN prediction model, the accuracy also improved by 6%. In addition, we used this model to predict the birthweight classification (SGA, AGA and LGA), and the obtained results were better than those of ML methods.

## Methods

Traditionally, the estimation of fetal weight in China is generally based on existing regression models that use multiple parameters established by foreign scholars. As a result, due to the individual differences in different populations, the use of these methods to estimate the weight of a fetus in China will result in large errors, especially for large especially for large or low-weight children. Hybrid-LSTM model has obtained information from experience and mine complex concepts implicit in experience to make more efficient and reasonable decisions.

### The proposed birthweight prediction framework

**Fig. 1:**
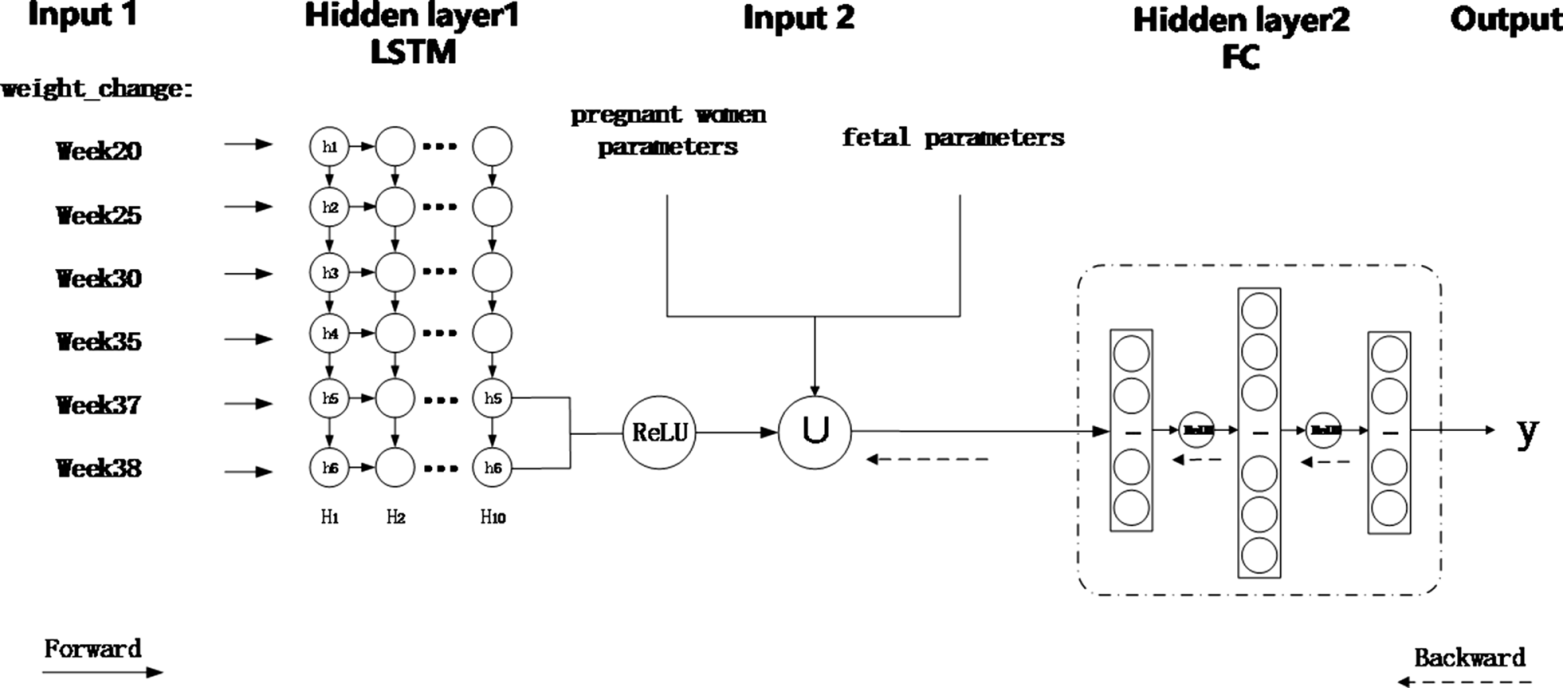
The proposed birthweight prediction based on Hybrid-LSTM

This study proposed to predict the birthweight using temporal prediction techniques. The block diagram of the prediction process is shown in Fig.1.

Overall, the input predictor set of temporal prediction model is comprised of pregnant women parameters, the fetal parameters and the weight change series. The relevant physiological fetal parameters are obtained via fetal ultrasonography, while the pregnant women parameters and the weight change series are obtained from the obstetric electronic medical records.

However, the frequency of antenatal examinations differs according to the different health conditions of pregnant women. In order to analyze the data, we need to standardize them in both time and magnitude dimensions. Therefore, data preprocessing is a key step toward improving the data quality. During the preprocessing, we processed the missing data and standardized the features of the raw data to form a continuously Hybrid-LSTM prediction model of the antenatal examination. The input layer is composed of two branches, two of which are combined as the input of the fully connected (FC) layers (Hidden layer 2). The activation function between the layers is the ReLU activation function, and then the output layer is a single neuron prediction. Finally, the predictive models quality was validated by standard concepts of accuracy as well as clinical usefulness.

### The predictors and the time series measured data

The historical clinical data of pregnant women were saved in the electronic medical records (EMR) and the laboratory information management (LIS) systems. For this study, we used the ID card of pregnant women as the main index and extracted the inspection data from the beginning of pregnancy until the women gave birth. Then, the true birthweight and category (SGA, AGA, and LGA) of the newborn were used to form the output labels of the body weight predictors.

The model defines Y as a set of true birthweights and X as a model input parameter set. The input parameter set consists of 16 parameters, X = {{*h*, *w*, *a*, *uh*, *acp*}, {*fl*, *acf*, *bpd*, *hc*, *afi*}, {$weight\_change_t$}}, which correspond to the pregnant women parameters, the fetal parameters and the weight change series, respectively. Among them, the parameter set {*h*, *w*, *a*, *uh*, *acp*} represents the height, pre-pregnancy weight, pregnancy age, uterine height and abdominal circumference of pregnant women, respectively; {*f*, *acf*, *bpd*, *hc*, *afi*} represents the femur length, abdominal circumference, biparietal diameter, head circumference and amniotic fluid index of the fetus. Table 1. List of parameters used in the prediction model. {$weight\_change_t$} represents the weight change series of pregnant women (the weight change refers to the value of the current weight minus the pre-pregnancy weight of pregnant women) at 6 different time points during pregnancy that are the $20{\rm th}$, $25{\rm th}$, $30{\rm th}$, $35{\rm th}$, $37{\rm th}$ and $38{\rm th}$ weeks. The denotations of the parameters are shown in Table 1.

**Table 1:** List of parameters used in the prediction model

	Predictors	Notation	Predictors	Notation
Static predictors	h	Height (cm)	w	Initial Weight (kg)
	a	Age	uh	Uterine Height (cm)
	acp	Abdominal Circumference of pregnant women (cm)	fl	Femur Length (cm)
	acf	Abdominal Circumference of fetal (cm)	bpd	Biparietal Diameter (cm)
	hc	Head Circumference (cm)	afi	Amniotic Fluid Index (cm)
Time series predictors	$weight\_change_t$	Weight Change at $20{\rm th}$, $25{\rm th}$, $30{\rm th}$, $35{\rm th}$, $37{\rm th}$ and $38{\rm th}$ weeks)		

As a time-series processing recurrent neural network (RNN), LSTM is suitable for processing and predicting the events with relatively long intervals and delays in the time series. Additionally, it can solve the gradient explosion problem when RNNs are used for long-term sequence predictions.

**Fig. 2:**
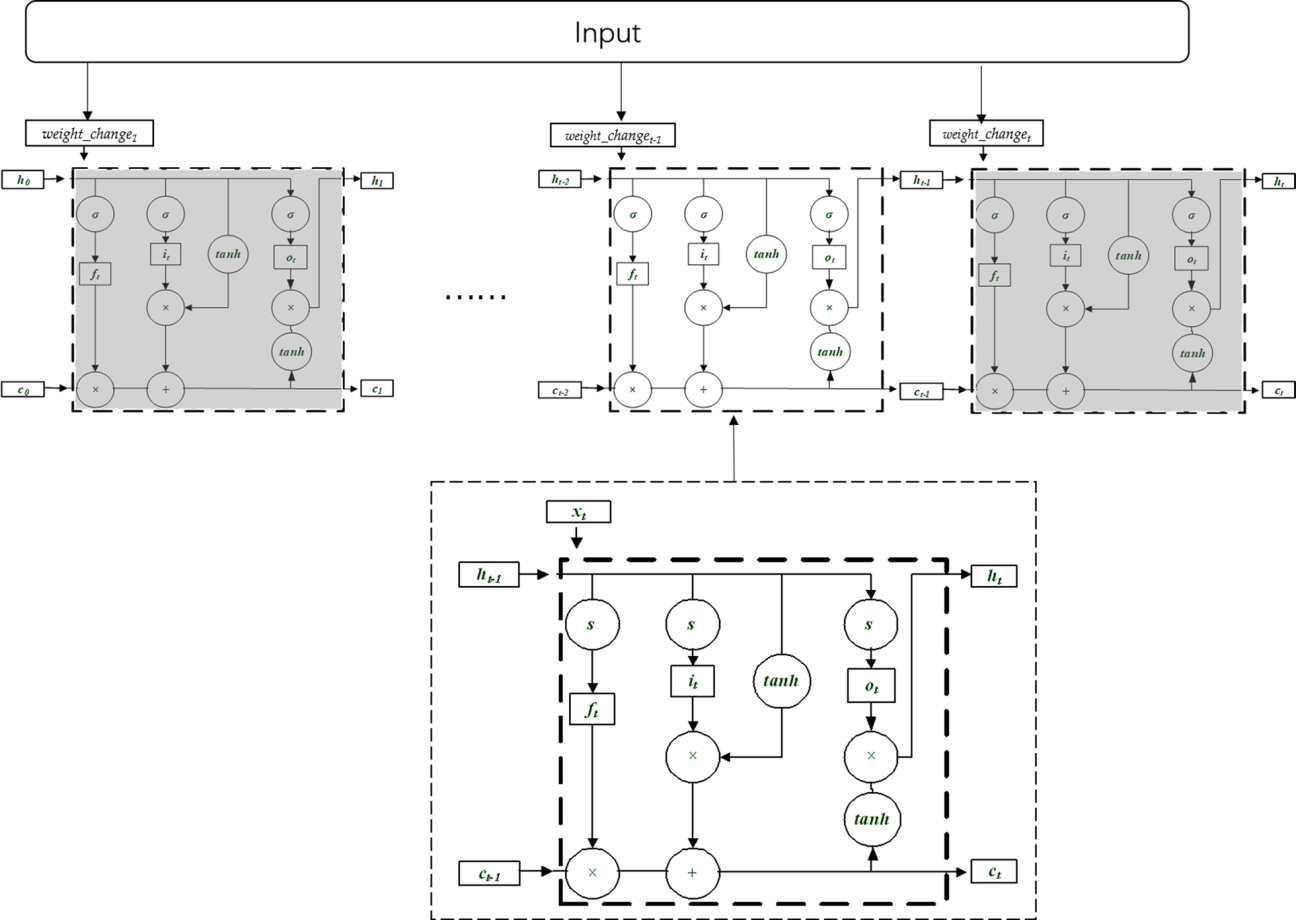
LSTM neuron structure

Each neuron of LSTM contains three control gates: the input gate, forget gate and output gate. The output at the previous time step enters the LSTM unit, and is then judged whether it is useful according to the cell. Only the useful information is kept, and the rest is forgotten at the forget gate.Equations (1) through (5) represent the parameter update process, where $\sigma$ represents the sigmoid function, $h_{t-1}$ represents the output of the LSTM at the previous time step, and $h_t$ represents the current output. The input gate, the forget gate and the output gate in the LSTM unit are defined as *i*, *f*, and *o*, respectively. $c_t$ is the state of the memory unit at the current time step, and Equation (4) represents the process of the state transition of the memory unit. The current state is calculated by the previous time step state, $c_{(t-1)}$ and the result of the forget gate and the input gate of the current time LSTM unit. In the data of this paper, the {$weight\_change_t$} represents the current input $x_t$. The structure of the LSTM unit is shown in Fig. 2.

1 $$\begin{aligned} i_{t}= & {} \sigma \left( {\varvec{W}}_{x i} x_{t}+{\varvec{W}}_{h i} h_{t-1}+b_{i}\right) \end{aligned}$$

2 $$\begin{aligned} f_{t}= & {} \sigma \left( {\varvec{W}}_{x f} x_{t}+{\varvec{W}}_{h f} h_{t-1}+b_{f}\right) \end{aligned}$$

3 $$\begin{aligned} o_{t}= & {} \sigma \left( {\varvec{W}}_{x o} x_{t}+{\varvec{W}}_{h o} h_{t-1}+b_{o}\right) \end{aligned}$$

4 $$\begin{aligned} {C}_{t}= & {} {f}_{t}{c}_{{t}-1}+ {i}_{ {t}} tanh \left( {W}_{{xc}} \ {x}_ {t}+ {W}_{hc} {h}_{{t}-1}+ {b}_ {c}\right) \end{aligned}$$

5 $$\begin{aligned} h_{t}= & {} o_{t} tanh \left( c_{t}\right) \end{aligned}$$

### Hybrid-LSTM model

#### Data preprocessing

Due to the variability of pregnancy check-ups date and the irregular inspection times, some loss will occur in the {$weight\_change_t$}. This is a practical problem that exists in the forecasting model, which is based on historical inspection data. In this work, {$weight\_change_t$} with more than 20% missing values were excluded from further analysis. We used a regression method to fill in the missing weight values with a regression algorithm based on the existing weight data. Then, we screened the pregnant women with more than five antenatal examinations and computed their weight values at different times in their pregnancies (in weeks) using the regression method fitted by a quadratic fitting function. Given the data sequence $(x_i, y_i)$, assume $x_i$ to be the number of pregnancy weeks, $y_i$ to be the weight change at $x_i$ and *P*(*x*) to be a quadratic fit function, the mean square error will then be calculated between the fitted function and the actual body weight sequence as follows:

6 $$\begin{aligned} \sum _{i=1}^{m}\left( P\left( x_{i}\right) -y_{i}\right) ^{2} \end{aligned}$$

The parameters of the quadratic fitting function are obtained by finding the minimum value of (1). In this study, we randomly selected 1000 sample data items for the fitting function experiment. The average relative error rate of the fitting result was 2.14%. Using this method, we acquired a set of maternal body weight sequences {$weight\_change_t$}, and the missing value processing was completed.

After processing the missing value, a model input parameter set was obtained. However, since different physiological parameters have different units and orders of magnitude, and in order to eliminate the impact of different units and data levels on the prediction results of the model, the data were normalized before the input of the parameters into the network model to ensure that each parameter had the same order of magnitude [12–14]. Standardization involved the calculation method shown in Equation (2), where *x* represents the current eigenvalue, $x_{min}$ and $x_{max}$ represent the minimum and maximum values of the current eigenvalue, respectively, *y* is the normalized eigenvalue and the normalized data range is [-1, 1].

7 $$\begin{aligned} y=\frac{2\left( x-x_{\min }\right) }{x_{\max }-x_{\min }}-1 \end{aligned}$$

#### Structure of the hybrid-LSTM prediction model

The structure of the hybrid-LSTM neural network is shown in Fig. 1. The input layer is composed of two branches, each branch is used to receive different types of the characteristic predictors. Input 1 is the weight_change data, which consists of six different weight changes. This input is the input of the LSTM network (Hidden layer1), which has 6 input and 2 output dimensions. The number of layers that are superimposed on the LSTM network is 10. After obtaining the output of the LSTM layers, the multibranch input layer is used to divide the physiological parameters into different categories, then the model merges with the related pregnant women and fetal physiological parameters and uses them as the input for several FC layers (Hidden layer2). The final output layer is a single neuron prediction. The multibranch input layer is used to divide the physiological parameters into different categories.

Regarding the activation function between the layers, the activation function between the layers was the ReLU activation function, except for the hidden layer connected to the output layer at which we used the linear activation function.

According to the calculations at the LSTM unit in the previous section, the results of providing the weight change sequence as Input1 of the LSTM are the outputs $h_5$ and $h_6$ at time $H_{10}$, recorded as $x_{h5}$, $x_{h6}$, respectively. The fetal parameters and those of the pregnant women are recorded as *x*_*other*_. Equation (8) represents the construction of the input parameters of the fully connected layer, and Equations (9) and (10) represent the input and output processing of the fully connected layer of each layer of the model, where *m* is the number of current fully connected layer nodes (including the input layer), $w_{ij}$ is the weight between node *i* and node *j*, $b_j$ is the threshold of node *j* and the output value of each node is $y_j$, which is also the next *x* of the fully connected layer; then, the final output of the model is the value of *y* when *j* is the last output layer node.

8 $$\begin{aligned} x_{{init}}= & {} ReLU\left( x_{h 5}, x_{h 6}\right) \cup \left( x_{{other}}\right) \end{aligned}$$

9 $$\begin{aligned} S_{j}= & {} \sum _{i=0}^{m-1} w_{i j} x_{i}+b_{j} \end{aligned}$$

10 $$\begin{aligned} y_{j}= & {} ReLU\left( S_{j}\right) \end{aligned}$$

### Parameter settings of the hybrid-LSTM

The Hidden layer2 includes 3 fully connected layers with the dimensions of $12 \times 20\,\times \,12 \times 1$. The number of neurons in the second layer is a relatively important factor that affects the convergence. Therefore, in this paper, we compare the effects of different dimensions by comparing different experiments while using the same data set and performing the same number of training epochs each time. The number of the second fully connected layer neurons is determined by comparing the mean square error (MSE), as shown in Equation (11).

11 $$\begin{aligned} M S E=\frac{1}{m} \sum _{i=1}^{m}\left( y_{i}-{\hat{y}}_{i}\right) ^{2} \end{aligned}$$

The best number of the second fully connected layer neurons is selected when the minimum MSE appears. The hybrid-LSTM neural network training has two stop conditions. The first condition is to reach the maximum number of epochs. The second condition is to have the error of 10 consecutive cycles less than the predetermined minimum error. Benefit from these two stop conditions, any unnecessary training time in the model can be reduced, and the overfitting phenomenon can be effectively prevented .

### Evaluation metrics for birthweight prediction

In this study, we used two indicators to evaluate the performance of the proposed forecasting model. The first one is the mean relative error (MRE). This index can be used as a common evaluation standard in regression analysis, which well reflects the prediction accuracy and performance of the prediction model. It can be calculated as follows:

12 $$\begin{aligned} M R E=\frac{1}{n} \sum _{i=1}^{n}\left| \frac{{\hat{y}}-y}{y}\right| \end{aligned}$$

where *y* is the true value of the sample, ${\hat{y}}$ is the predicted value of the model and n is the total sample size.

The accuracy criterion is also considered together with MRE. Accuracy means that we will judge the prediction result to be correct when the error between the predicted and actual fetal body weight is within ± 250 grams, which is acceptable for clinicians [15]. Accuracy can be calculated as Equation (13).

13 $$\begin{aligned} Accuracy=\frac{1}{n} \sum _{i=1}^{n} bool(|{\hat{y}}-y|<250) \end{aligned}$$

where *y* is the true value of the sample, ${\hat{y}}$ is the predicted value of the model and *n* is the total sample size

### The evaluation metric for birthweight classification

The classification of the birthweight range has different standards in different countries and regions. This paper is based on the evaluation criteria for Eastern China. We identify a fetus with a birthweight of less than 2500 grams as small for gestational age (SGA), a fetus with a birthweight greater than 4000 grams as large for gestational age (LGA), and a fetus with a birthweight value between these two values as appropriate for gestational age (AGA).

SGA and LGA fetuses have potential risks of diseases; thus, it is practical to accurately predict the category of the fetus before delivery. We added a one-step classification operation for the model’s predictions with the categories of SGA, AGA and LGA, we then labeled them with different numbers according to the specified weight range. Equation (14) is a description of the classification accuracy rate.

14 $$\begin{aligned} Category \ Accuracy =\frac{1}{n} \sum _{i=1}^{n} bool ({\hat{y}}=y) \end{aligned}$$

where *y* is the true value of the sample, ${\hat{y}}$ is the predicted value of the model and *n* is the total sample size.

## Results and discussion

### Dataset

The dataset used in the paper comes from a hospital in Eastern China, which has a huge amount of data such as the maternal ultrasound records, birth check reports and imaging data. In this experiment, we selected 5,759 qualified samples from the obstetric electronic medical records between January 1, 2018 and October 1, 2018. The statistical information of pregnant women parameters, the fetal parameters and the weight change series is summarized in Table 2.

**Table 2:** Summary statistics of pregnant women parameters, the fetal parameters and the weight change series in China, 2018.1-2018.10

	Mean	Standard deviation	Minimum	Median	Maximum
weight_change20w (kg)	58.2	8.1	38.5	57.5	116.5
weight_change25w (kg)	61.3	8.4	39.0	60.0	130.5
weight_change30w (kg)	64.0	8.3	40.5	63.0	132.0
weight_change35w (kg)	66.5	8.4	42.5	65.5	134.0
weight_change37w (kg)	67.6	8.5	43.5	66.8	162.0
weight_change38w (kg)	68.6	8.6	44.0	67.9	158.8
age	29.3	4.2	18.0	29.0	46.0
height (cm)	160.4	4.8	144.0	160.0	184.0
initial weight (kg)	54.7	8.1	35.0	54.0	163.0
acp (cm)	100.2	6.0	73.0	100.0	145.0
uh (cm)	34.4	1.9	26.0	34.0	73.0
bpd (cm)	9.3	0.3	7.9	9.3	10.5
hc (cm)	33.2	1.0	29.3	33.2	39.2
acf (cm)	34.1	1.5	28.9	34.1	41.7
fl (cm)	7.2	0.2	5.9	7.2	8.2
afi	10.6	3.6	2.0	10.3	29.6
birthweight (g)	3399.1	385.8	1520.0	3390.0	4950.0
parity	0.4	0.5	0	0	3.0
delivery weeks	39.4	0.9	37.0	39.0	44.0

For the purpose of training the machine learning models and setting the parameters, we split the samples randomly into a training set and a test, with a 80% vs 20% ratio. Five-fold cross-validation was adopted in the evaluation experiments.

### Comparison of machine learning prediction models

In order to more effectively verify the experimental results of this model, we compared the prediction results of the calculation formula, shown in Equation (15), with those of SVR and BPNN and compared the experimental results based on different births and delivery weeks.

The calculation formula uses the Zhuo’s Formula, which has a relatively good prediction results compared with calculation formula [5]. In addition large sample studies have proved that Zhuo’s Formula has clinical significance without distinguishing LGA, SGA and AGA [4], as follows:

15 $$\begin{aligned} G=u h * 100 \end{aligned}$$

where *G* is the predicted weight, and *uh* is the uterine height.

The machine learning method that we used is the SVM regression (SVR), which has been widely applied in medical diagnostics with small sample sets, the BPNN is the standard 3-layer neural network, Linear regression(Linear-R) is a statistical analysis method to determine the quantitative relationship among multiple variable, the CNN is a kind of feed forward neural networks with depth structure and convolution computation, and the RF is a decision tree algorithm based on bagging ensemble.

Based on the above-mentioned features in Table 3, we constructed these machine learning models to predict the birthweight. Using the optimal parameters for each model, the predictive models were corroborated via a validation set which was derived from the training dataset by 5-fold cross-validation.

**Table 3:** Summary of parameter values in each model

Models	Parameters	Values	Parameters Meaning
SVM	kernel	linear	kernel function
	C	1.0	regularization parameter
	Cache_size	200	specify the size of the kernel cache
	tol	0.001	tolerance for stopping criterion
	gamma	scale	kernel coefficient
BPNN	kernel initializer	uniform	kernel initializer function
	activation1	relu	activation of hidden layer
	activation2	sigmoid	activation of output layer
	optimizer	Adam	training optimization algorithm
	epochs	200	number of times shown to the network
	batch size	128	batch size
Linear-R	fit_intercept	True	whether to calculate the intercept for this model
	normalize	False	whether to standardize the data
	copy_X	True	If True, X will be copied
CNN	lr	0.01	learning rate
	epochs	100	number of times shown to the network
	optimizer	Adam	training optimization algorithm
	Conv1_in_channels	1	number of channels in the input image
	Conv1_outchannels	10	number of channels produced by the convolution
	Conv1_kernel_size	1	size of the convolving kernel
	Conv1_strid	2	stride of the convolution
	Conv2_in_channels	10	number of channels in the input image
	Conv2_outchannels	20	number of channels produced by the convolution
	Conv2_kernel_size	1	size of the convolving kernel
	Conv2_strid	2	stride of the convolution
RF	n estimators	200	the number of trees in the forest
	Min_samples_leaf	1	the minimum number of samples required to be at a leaf node
	Min_samples_split	2	the minimum number of samples required to split an internal node
	max depth	None	the maximum depth of the tree.
	max features	7	the number of features to consider when looking for the best split

The corresponding MSE values after training the different network structures are shown in Table 4, when the number of neurons in the layer is 20, the network performance is the best. As we continue to increase the number of hidden layer neurons, the network performance does not improve. Therefore, the number of the second FC layer neurons was determined to be 20.

**Table 4:** MSE after the training of different network structures

Number of second FC layer neurons	MSE
12	0.0553
14	0.0424
16	0.0281
18	0.0212
20	0.0146
22	0.0217
25	0.0256
30	0.0319

The model’s parameter settings for the dropout [16, 17] were used between the hidden layers to prevent the model from overfitting, and the dropout parameter was set to 0.2. Meanwhile, the batch size was set to 50. The model used the mean square error (MSE) as the loss function with Adam [18] as the optimizer.

#### Overall comparison results

First, we compared the prediction effects of different methods based on all the data. The average values of the results after 5-fold CV are shown in Table 5.

**Table 5:** Prediction average results of different methods after 5-fold CV

Method	MRE (%)	Accuracy (%)
Zhuo’s Formula	8.52 ± 0.4	58.7
SVM-R	7.33 ± 0.4	65.5
BPNN	6.36 ± 0.4	73.0
Linear-R	5.77 ± 0.4	70.4
CNN	9.21 ± 0.4	51.0
RF	5.86 ± 0.4	69.9
Hybrid-LSTM	5.65 ± 0.4	79.2

As shown in Table 5, Our study found that the hybrid-LSTM prediction model, outperformed Calculation formul, SVR, BPNN, Linear-R, CNN and RF, achieved average accuracy of $> 0.79$, and the error of the hybrid-LSTM prediction model for the birthweight is controlled within 5.7%, and the results of this method are far superior to several other prediction methods. This suggests that hybrid-LSTM prediction models have better generalization capabilities compared to other models for predicting the actual fetal body weight. Even though Linear-R and RF also has lower MRE, this might not be acceptable as lower accuracy means that these model might not perform well in AGA. The results of our study indicate that hybrid-LSTM prediction model are well suited for the prediction of fetal weight within acceptable error range in most people.

#### Performance comparison by delivery weeks

In addition, to compare the effects of different delivery weeks and parities on the predictive effect, we divided the data into the first and multiple deliveries. On this basis, the data were divided into the groups of less than 39 weeks, 39 weeks, 40 weeks and more than 40 weeks of delivery.

**Table 6:** Prediction results for different periods

Delivery		Less than 39 weeks		39 weeks		40 weeks		More than 40 weeks	
Evaluation		MRE	Accuracy	MRE	Accuracy	MRE	Accuracy	MRE	Accuracy
First delivery	Formula	8.3	59.5	8.9	54.5	8.7	56.3	8.8	52.3
	SVM	7.2	68.7	7.3	67.0	7.4	68.0	7.6	65.4
	BPNN	6.2	75.0	6.7	72.0	6.5	70.5	6.2	71.2
	Linear-R	6.0	70.1	5.8	68.4	6.1	67.0	5.8	69.2
	CNN	7.3	63.6	8.8	51.6	9.5	49.8	9.8	51.4
	RF	5.9	75.3	5.4	70.7	5.9	66.2	6.1	65.4
	Hybrid-LSTM	5.11	84.8	6.0	76.7	5.7	79.3	5.7	81.0
Multiple deliveries	Formula	7.9	61.0	8.6	56	8.1	60.5	9.2	51.5
	SVM	6.9	70.3	7.2	62	7.6	67.0	7.6	63.0
	BPNN	6.5	75.7	6.3	73	6.7	71.0	6.8	68.2
	Linear-R	5.9	69.2	6.0	72.4	5.5	68.7	7.9	61.1
	CNN	10.1	43.8	8.3	53.5	10.1	46.3	8.7	44.4
	RF	7.4	61.5	5.8	70.6	6.5	67.2	7.2	66.7
	Hybrid-LSTM	5.3	81.6	5.7	78.6	6.1	74.8	7.3	69.0

As shown in Table 6, our proposed method achieved better prediction results than the other methods for different births and delivery weeks. However, regarding the data for multiple delivery women with more than 40 weeks of delivery, the prediction results were poor and were 10 percentage points lower than the average. The prediction was the best for the data of single-child pregnant women for less than 39 weeks, reaching approximately 85%. This shows that the relationship between the birthweight and the parameters of pregnancy is more stable for first delivery women than for multiple delivery ones. More than 37 weeks of delivery is a medically recognized full-term fetus. The results of this experiment showed that the predicted birthweights are more accurate and reliable as the delivery time is more within the normal range.

#### Comparion of birthweight classification results

We compared our model with the Zhuo’s Formula [5], the logistic regression (LR) method [7] and the BPNN, CNN and RF as shown in Table 7.

**Table 7:** Prediction results of different methods

Method	Accuracy (%)
Formula	78.0
LR	85.5
BPNN	89.0
CNN	90.6
RF	91.6
Hybrid-LSTM	93.3

Table 7 shows that the model exhibited a good ability to predict the classification of the birthweight, which does not only reduce the risk of fetal diseases but can also help the doctors to make appropriate clinical decisions and improve the pregnancy success rate.

## Conclusion

In the paper, we proposed a hybrid birthweight prediction model based on LSTM, which establishes a continuous model of the parameters related to the pregnant women and fetal physical examination. The experimental results show that the proposed birthweight prediction model does not only increase the model convergence rate but also improves the birthweight prediction accuracy by 6%. There is room for improvement in the deep neural network model to improve the accuracy and practicality of the model prediction. Additionally, this study explored the risk classification of different categories of the birthweight, and we obtained better accuracy than other methods, which can be useful for clinical applications.

## Data Availability

The data that support the findings of this study are available from Hangzhou Women’s Hospital, but restrictions apply to the availability of these data, which were used under license for the current study, and so are not publicly available. Data are however available from the authors upon reasonable request and with permission of Hangzhou Women’s Hospital.

## Abbreviations

LSTM: Long short-term memory
MRE: Mean relative error
SGA: Small-for-gestational-age
LGA: Large-for-gestational-age
AGA: Appropriate-for-gestational-age
CNN: Convolutional neuron network
RF: Random forest
SVR: Support vector regression
LR: Logistic regression
BPD: Biparietal diameter
AC: Abdominal circumference
HC: Head circumference
FL: Femur length
ML: Machine learning
ANN: Artificial neural network
DBN: Deep belief network
FC: Fully connected
LIS: Laboratory information management
EMR: Electronic medical records
RNN: Recurrent neural network
MSE: Mean square error
CV: Cross-validation
